# Visual mnemonics for serum protein electrophoresis

**DOI:** 10.3402/meo.v18i0.22585

**Published:** 2013-09-24

**Authors:** Carlos E. Medina-De la Garza, Marisela García-Hernández, María de los Ángeles Castro-Corona

**Affiliations:** 1 Immunology Department, School of Medicine, Universidad Autónoma de Nuevo León, Monterrey, Mexico; 2 Center for Research and Development in Health Sciences (CIDICS), Universidad Autónoma de Nuevo León, Monterrey, Mexico; 3 Biochemistry and Molecular Medicine Department, School of Medicine, Universidad Autónoma de Nuevo León, Monterrey, Mexico

Mnemonics are systems to improve and assist memory. These learning techniques are widely used in different settings; its name was derived from the Greek goddess of memory, Mnemosyne, daughter of Gaia and Uranus. Medicine learning is no exception for use of these tools (1). Mnemonics based in letters and word listings, however, have natural limitations because the language used may not be of general knowledge and therefore not at general reach. On the contrary, visual mnemonics may have a broader reach and be useful regardless of language or cultural background.

Since its description and introduction to clinical practice, serum protein electrophoresis has been useful as a first-line test for serum albumin and globulins and to identify monoclonal gammopathies, agammaglobulinemia, polyclonal production of immunoglobulins, and increase/decrease of other proteins (2). The electrophoresis drawing provided by laboratory shows five main protein fractions of serum. Nevertheless, it is sometimes difficult for students, residents, nurses, and non-specialized medical practitioners to remember and recognize these fractions. After dealing with this difficulty with medical students and residents, we propose here an easy to remember, visual mnemonics for this purpose. In Figure 1, “A” shows the schematic representation of a normal serum protein electrophoresis densitometric scanning with the albumin fraction α-1, α-2, β, and γ peaks. By holding the right hand as shown “B”, we can recall the shape of the normal electrophoresis, from which we can derive changes in its profile indicating abnormality, mainly in clinically relevant gamma fraction (i.e., a spike for monoclonal gammopathies, a broad peak for polyclonal gammopathies or absence for agammaglobulinemia) (2). Although this hand-aided outline is by no means a diagnostic mnemonics for itself, to recall the normal profile and the five main peaks of serum protein electrophoresis scanning is the first step to detect changes/anomalies and alert for further testing and diagnosis by proper techniques.

**Fig. 1:**
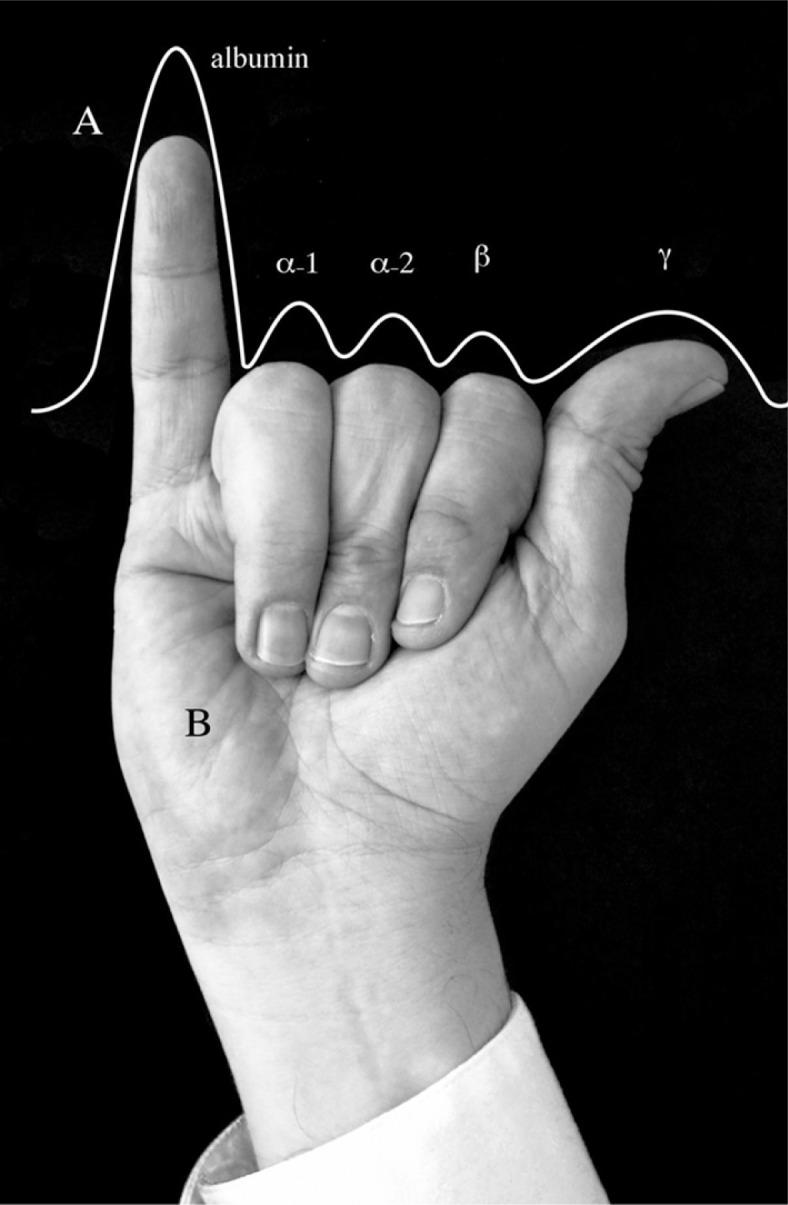
Right-hand position for serum protein electrophoresis mnemonics.

## References

[1] Yousaf S, Chaudhry M (2006). Mnemonics for medical undergraduates.

[2] O'Connell TX, Horita TJ, Kasravi B (2005). Understanding and interpreting serum protein electrophoresis. Am Fam Physician.

